# Complete Coding Sequence of a Novel Bluetongue Virus Isolated from a Commercial Sheeppox Vaccine

**DOI:** 10.1128/MRA.01539-19

**Published:** 2020-03-05

**Authors:** Paulina Rajko-Nenow, Natalia Golender, Velizar Bumbarov, Hannah Brown, Lorraine Frost, Karin Darpel, Chandana Tennakoon, John Flannery, Carrie Batten

**Affiliations:** a The Pirbright Institute, Pirbright, Surrey, United Kingdom; b Department of Virology, Kimron Veterinary Institute, Bet Dagan, Israel; Portland State University

## Abstract

The full genome sequences of two isolates of bluetongue virus (BTV) from a commercial sheeppox vaccine were determined. Strain SPvvvv/02 shows low sequence identity to its closest relative, strain BTV-26 KUW2010/02, indicating the probable detection of a novel BTV genotype, whereas strain SPvvvv/03 shows high sequence identity to strain BTV-28/1537/14.

## ANNOUNCEMENT

Bluetongue (BT) is a disease of ruminants which is transmitted by blood-feeding *Culicoides* midges (1). BT is one of the major diseases of ruminants listed by the World Organisation for Animal Health (OIE), and suspicion of disease needs to be reported to veterinary authorities. BT virus (BTV) belongs to the *Orbivirus* genus, and its genome consists of 10 linear double-stranded RNA segments encoding seven structural (VP1 to VP7) and five nonstructural (NS1 to NS5) proteins (2, 3). While the typical BTV genotypes (BTV-1 through BTV-24) are noncontagious and almost exclusively transmitted via their biological insect vector, *Culicoides* biting midges, direct transmission between infected animals has been documented for the atypical genotypes BTV-25, BTV-26, BTV-27, and BTV-28 (4–7).

BTV contamination of commercial batches of sheeppox and lumpy skin disease vaccines was previously reported (8). However, full-genome sequencing data were incomplete in this study and were limited to only a few segments. A subsequent study released the full genome sequence and demonstrated that the sheeppox vaccine-derived BTV-28 strain (BTV-28/1537/14) caused clinical signs in experimentally infected ewes and could be directly transmitted between infected and healthy sheep (7).

The commercial sheeppox vaccine (JOVAC, batch number 200214/01) was resuspended in 1 ml phosphate-buffered saline (PBS; pH 7.20) and used as the inoculum for virus propagation in baby hamster kidney (BHK) cells. Total RNA was extracted from the cell pellets using TRIzol reagent (Life Technologies, UK). Single-stranded RNA (ssRNA) was removed by RNase T1 digestion, and then double-stranded DNA (dsDNA) synthesis was performed using SuperScript III reverse transcriptase (RT) (Life Technologies) and the NEBNext Ultra II nondirectional RNA second-strand synthesis module (New England Biolabs, UK) according to the manufacturer’s instructions. Library preparation was performed using the Nextera XT DNA library kit (Illumina, USA), and paired-end read sequencing (2 × 150 bp) was carried out using an Illumina MiSeq instrument. The raw data were quality (with the parameter -q 25) and adapter trimmed along with the removal of short sequences (<50 bp) using Trim Galore (9). Paired-end reads were mapped to a set of reference genomes using Bowtie2 version 2.2.9 (10) with the “relax” setting to increase sensitivity. Local alignment was performed using a short seed (–15), allowing for one mismatch in the seed. The DiversiTools software (11) was used to generate the consensus sequence. Subsequently, the best consensus sequences (without any gaps) were used as the reference sequence (Table 1) for each segment, and the reads were mapped using BWA-MEM version 0.7.12-r1039 (12). In addition, the consensus sequences generated from BWA-MEM mapping were compared to those generated using in-house *de novo* mapping (unpublished protocol), but no changes were identified.

**TABLE 1:** Sequencing data for isolates SPvvvv/02 and SPvvvv/03

Isolate^*a*^	GenBank accession no.	Segment no.	Length (bp)	No. of mapped reads	Avg depth (bp)	Reference sequence name	Reference sequence accession no.	Nucleotide identity (%)
**SPvvvv/02**	MN723870	1	3,944	202,361	7,104	BTV-26 KUW2010/02	JN255156	92.7
	MN723871	2	2,928	142,992	6,746	BTV-26 KUW2010/02	HM590642	72.5
	MN723872	3	2,766	136,532	6,711	TUN2017	MF124284	99.7
	MN723873	4	1,982	77,604	5,311	TUN2017	MF124285	100.0
	MN723874	5	1,768	63,587	4,885	BTV-28/1537/14	MH559812	98.1
	MN723875	6	1,629	73,353	6,157	BTV-26 KUW2010/02	JN255159	88.0
	MN723876	7	1,157	25,474	2,912	BTV-26 KUW2010/02	HM590644	91.8
	MN723877	8	1,121	23,376	2,742	BTV-28/1537/14	MH559810	98.1
	MN723878	9	1,064	37,481	4,400	TUN2017	MF124290	99.8
	MN723879^*b*^	10	822	7,999	1,272	BTV-26 KUW2010/02	JN255162	87.2
**SPvvvv/03**	MN723880	1	3,944	440,731	15,666	BTV-28/1537/14	MH559813	99.9
	MN723881	2	2,925	264,358	12,595	BTV-28/1537/14	MH559807	99.9
	MN723882	3	2,773	342,225	16,928	BTV-28/1537/14	MH559808	100.0
	MN723883	4	1,982	199,951	13,802	BTV-28/1537/14	MH559814	100.0
	MN723884	5	1,766	186,852	14,099	BTV-28/1537/14	MH559812	99.9
	MN723885	6	1,639	316,536	26,612	BTV-28/1537/14	MH559815	100.0
	MN723886	7	1,157	55,272	6,213	BTV-28/1537/14	MH559811	100.0
	MN723887	8	1,121	63,581	7,428	BTV-28/1537/14	MH559810	100.0
	MN723888	9	1,064	79,039	9,272	BTV-28/1537/14	MH559816	99.9
	MN723889^*b*^	10	822	12,042	1,866	BTV-28/1537/14	MH559809	100.0

a The entire genome of SPvvvv/02 (segments 1 through 10) has a segment length of 19,181 bp, 790,759 mapped reads, and an average coverage depth of 4,824 bp, and the entire genome of SPvvvv/03 (segments 1 through 10) has a segment length of 19,193 bp, 1,960,587 mapped reads, and an average coverage depth of 12,448 bp.

b BTV segment 10 (GenBank accession number KT946752) was first sequenced directly using RNA extracted from a commercial sheeppox vaccine (8); it had 100% nucleotide identity with SPvvvv/02 and 98.39% identity with both SPvvvv/03 and BTV-28/1537/14.

The full genome sequences of the two isolates, SPvvvv/02 and SPvvvv/03, were obtained from the sheeppox vaccine. SPvvvv/02 showed only 72.5% and 88.0% nucleotide identity to its closest relative, BTV-26 KUW2010/02, in segments 2 and 6, respectively (Table 1). This finding indicates the detection of a putative novel genotype of BTV. In contrast, SPvvvv/03 was highly identical to strain BTV-28/1537/14 across all 10 segments (99.86% to 100%), but its segment 1 was slightly shorter (3,944 bp) in comparison with that of BTV-28/1537/14 (3,985 bp). Our study indicates that the commercial sheeppox vaccine was contaminated with more than one novel BTV genotype.

### Data availability.

The full genome sequences of isolates SPvvvv/02 and SPvvvv/03 have been deposited in GenBank under accession numbers MN723870 through MN723879 and MN723880 through MN723889, respectively. The raw sequencing reads have been deposited in the NCBI SRA under BioProject accession number PRJNA599340.
